# β-Catenin signaling positively regulates glutamate uptake and metabolism in astrocytes

**DOI:** 10.1186/s12974-016-0691-7

**Published:** 2016-09-10

**Authors:** Victoria Lutgen, Srinivas D. Narasipura, Amit Sharma, Stephanie Min, Lena Al-Harthi

**Affiliations:** Department of Immunology and Microbiology, Rush University Medical Center, 1735 W Harrison Street, 614 Cohn, Chicago, IL 60612 USA

**Keywords:** Excitatory amino acid transporter 2 (EAAT2), Glutamine synthetase (GS), Excitotoxicity, Glia, Neuroinflammation, β-Catenin

## Abstract

**Background:**

Neurological disorders have been linked to abnormal excitatory neurotransmission. Perturbations in glutamate cycling can have profound impacts on normal activity, lead to excitotoxicity and neuroinflammation, and induce and/or exacerbate impairments in these diseases. Astrocytes play a key role in excitatory signaling as they both clear glutamate from the synaptic cleft and house enzymes responsible for glutamate conversion to glutamine. However, mechanisms responsible for the regulation of glutamate cycling, including the main astrocytic glutamate transporter excitatory amino acid transporter 2 (EAAT2 or GLT-1 in rodents) and glutamine synthetase (GS) which catalyzes the ATP-dependent reaction of glutamate and ammonia into glutamine, remain largely undefined.

**Methods:**

Gain and loss of function for β-catenin in human progenitor-derived astrocyte (PDAs) was used to assess EAAT2 and GS levels by PCR, western blot, luciferase reporter assays, and chromatin immunoprecipitation (ChIP). Further, morpholinos were stereotaxically injected into C57BL/6 mice and western blots measured the protein levels of β-catenin, GLT-1, and GS.

**Results:**

β-Catenin, a transcriptional co-activator and the central mediator of Wnt/β-catenin signaling pathway, positively regulates EAAT2 and GS at the transcriptional level in PDAs by partnering with T cell factor 1 (TCF-1) and TCF-3, respectively. This pathway is conserved in vivo as the knockdown of β-catenin in the prefrontal cortex results in reduced GLT-1 and GS expression.

**Conclusions:**

These studies confirm that β-catenin regulates key proteins responsible for excitatory glutamate neurotransmission in vitro and in vivo and reveal the therapeutic potential of β-catenin modulation in treating diseases with abnormal glutamatergic neurotransmission and excitotoxicity.

## Background

The glutamate/glutamine cycle is an integral component of brain homeostasis. Neurons release glutamate, which is the most abundant neurotransmitter in the brain involved in learning, memory, and cognition. In excess, glutamate is neurotoxic and is linked to a number of neurodegenerative diseases such as Alzheimer’s disease [1], Parkinson’s disease [2], and neuroAIDS [3, 4]. Both astrocytes and microglia have specific transporters to clear extracellular glutamate and convert it into glutamine in a regulated process. Upon glutamate uptake, glutamine synthetase (GS) catalyzes the ATP-dependent reaction of glutamate and ammonia into glutamine. Glutamine is released and in turn is taken up by neurons for conversion back to glutamate by glutaminase [5].

Astrocytes have a dominant role in the glutamate/glutamine cycle. They clear >90 % of excess glutamate [5, 6] through excitatory amino acid transporters (EAATs) 1 and 2. Although EAAT1 and EAAT2 are primarily expressed on astrocytes, EAAT2 (or GLT-1 in rodents) is the major transporter of glutamate on astrocytes [7, 8]. The mechanism by which astrocytes regulate the expression of two key proteins in the glutamate/glutamine cycle, EAAT2 and GS, are not clearly understood. Studies have identified a role of nuclear factor kappa B (NF-kB) in the regulation of astrocytic EAAT2; however, NF-kB activation appears to have a bidirectional role. In mouse and human astrocytes, epidermal growth factor (EGF) stimulates NF-kB to induce EAAT2 expression [9–12]. However, in human astrocytes, TNF-α stimulation of NF-kB reduces EAAT2 protein expression [13]. Both scenarios require external stimulation of NF-kB activation suggesting a non-NF-kB-dependent regulatory pathway for baseline conditions of EAAT2 protein expression. Of note, although basal (e.g., without an inducing signal) expression of EAAT2 in rodent-derived astrocytes is little to undetectable [9, 12], in human-derived astrocytes and astroglioma cell lines, EAAT2 expression is measureable making these cells ideal to study agents capable of down-modulating baseline EAAT2 [7, 8, 14]. Regulation of astrocytic GS remains less defined although studies have indicated GS regulation by transient molecules including extracellular signals, inflammatory mediators, and hormones [13, 15–17]. Studies of rat liver epithelial cell line RL-ET-14, mouse hepatocellular carcinoma, and human pancreatic tumors have revealed β-catenin regulation of EAAT2/GLT-1 and GS [15, 18, 19]. Collectively, these studies reveal the complex and highly regulated nature of glutamate cycling protein regulation under basal and inducible conditions.

Using microarray analysis, we previously showed that β-catenin knockdown in human progenitor-derived astrocyte (PDAs) leads to modulation of genes relevant in regulating aspects of glutamate neurotransmission [20]. This finding prompted further analysis of the direct role of β-catenin on key players in glutamate/glutamine cycling (EAAT2 and GS). β-Catenin is a central mediator of the Wnt/β-catenin pathway. In association with members of the T cell factor/lymphoid enhancer factor (TCF/LEF) family of transcription factors (TCF-1, TCF-3, TCF-4, LEF1), β-catenin functions as a transcriptional regulator of gene expression. β-Catenin also binds to cadherins to facilitate cell adhesion and cell-to-cell communication [21]. The Wnt/β-catenin pathway is engaged by Wnt ligands, small secreted glycoproteins, which bind to members of the seven-transmembrane G protein-coupled Frizzled receptors and co-receptor lipoprotein receptor-related protein 5 or 6 (LRP5/6). This induces a complex signaling cascade resulting in glycogen synthetase kinase 3β (GSK3β) destabilization to hypophosphorylate β-catenin and prevention of proteosomal degradation. Relocation of β-catenin to the nucleus allows either association with TCF/LEF for gene regulation or relocation to the cell membrane for cadherin binding.

We evaluated here the role of β-catenin in regulating the expression of EAAT2 and GS in human astrocytes and mouse medial prefrontal cortex (mPFC). We show that β-catenin positively regulates EAAT2 and GS expression but through association with different TCF/LEF members. In regulating EAAT2 and GS expression, β-catenin interacts with TCF-1 and TCF-3, respectively. These findings demonstrate that the β-catenin pathway is a critical regulator of EAAT2 and GS expression, which can be harnessed to remove excess glutamate in neurodegenerative diseases.

## Methods

### Cell culture

Progenitor-derived astrocyte (PDAs) were generated from neural progenitor cells (kindly provided by Dr. Eugene Major, NINDS, NIH, MD) as previously described [14]. Briefly, PDAs were maintained in progenitor medium consisting of Neurobasal media (Invitrogen) supplemented with 0.5 % bovine albumin (Sigma-Aldrich), neurosurvival factor (NSF; Lonza), N2 components (Invitrogen), 25 ng/ml basic fibroblast growth factor (bFGF), 20 ng/ml EGF (R&D Systems), 50 μg/ml gentamicin (Lonza), and 2 mM L-glutamine (Invitrogen). To induce differentiation, progenitor medium was replaced with PDA medium containing DMEM supplemented with 10 % heat-inactivated fetal bovine serum, 2 mM L-glutamine, and 50 μg/ml gentamicin. Cultures were >90 % positive for glial fibrillary acidic protein (GFAP) after 30 days of differentiation. Fetal-derived Normal Human Astrocytes (NHAs, Lonza) were maintained in Astrocyte Growth Media (AGM) BulletKit (Lonza) supplemented with 0.3 % heat-inactivated fetal bovine serum, 1 ml/ml ascorbic acid, 1 ml/ml rhEGF, 1 ml/ml AG-1000 (30 mg/ml gentamicin and 15 μg/ml amphotericin), 2.5 ml/ml insulin, and 10 ml/ml L-glutamine. Adherent primary cells were removed by treatment with 1 mM EDTA for 5 min with gentle scraping or pipetting multiple times.

### Plasmids, transfection, and small interfering RNAs (siRNAs)

The TOPflash plasmid (Millipore) detecting β-catenin signaling contains multiple TCF/LEF binding sites linked to a firefly luciferase reporter. An approximate 2.5-kb human EAAT2 promoter linked to firefly luciferase plasmid (EAAT2-prom) was a kind gift from Dr. Paul B. Fisher (Virginia Common University, Virginia, USA). A pcDNA plasmid harboring the constitutively active β-catenin gene termed as pABC (S33Y mutation; 19286) or control pcDNA (10792) was obtained from Addgene. Plasmids were transfected using Lipofectamine 2000 as per manufacturer’s instruction (Invitrogen). ON-TARGET plus SMART pool siRNAs specific for TCF-1 (L-019735-00), TCF-3 (L-014703-00), TCF-4 (L-003816-00), LEF1 (L-015396-00), β-catenin (L-003482-00), and scrambled (D-001810-10) all from Thermo Fisher Dharmacon were transfected using Lipofectamine siRNAmax (Invitrogen Life Technologies) according to the reagent protocol. Cells were approximately 70–80 % confluent at the time of transfection.

### Firefly luciferase reporter assay

Twenty-four hours post transfection, the culture medium was removed, cells were gently washed with PBS once, and 100 μl of passive lysis buffer (PLB) was added and incubated at 37 °C for 5 min. Cells were lysed by pipetting up and down several times and spun at 5000 rpm for 4 min to remove debris, and 10–20 μl was used to assay for luciferase activity using luciferase assay system (Promega) in a single injector luminometer. Total protein concentration was measured using bicinchoninic acid assay (BCA) protein assay kit (Thermo Fisher), and relative light units were normalized to micrograms of protein. Graphs were plotted from data obtained as a mean of three independent experiments with standard deviation as error bars.

### Western blotting

In vitro cell samples were lysed by incubating with 50 μl of PLB for 5 min at 37 °C and pipetting up and down several times. In vivo brain tissue samples were lysed by incubating with 100 μl Radioimmunoprecipitation Assay (RIPA) buffer and homogenized. Both lysates were cleared for cell debris by spinning at 5000 rpm for 5 min. Total protein content of the cleared lysate was estimated by BCA, and 5–20 μg of total protein was separated on a 10 % SDS PAGE, transferred to a nitrocellulose membrane, blocked with superblock (Thermo Fisher) containing 0.1 % Tween 20 (T20) for 1 h, and incubated with appropriate primary antibodies overnight at 4 °C in superblock-0.1 % T20. Membranes were washed extensively with Tris buffer saline containing 0.1 % T20 (TBST) and incubated with appropriate secondary antibody in Superblock-0.1 % T20 for 45 min at room temperature (RT). Membranes were again washed extensively in TBST and developed with SuperSignal West Femto Maximum Sensitivity Substrate (Thermo Fisher) according to instructions. Relative quantification of proteins were carried out using ImageJ software and normalized to GAPDH (Glyceraldehyde 3-phosphate dehydrogenase). Primary antibodies used were as follows: anti-rabbit β-catenin antibody (Sigma-Aldrich; C 2206), anti-mouse EAAT2 antibody (Cell Signaling; 3838), anti-mouse GS antibody (Thermo Fisher; PA5-29737), and anti-rabbit GAPDH antibody (Sigma-Aldrich; G9545). Secondary antibodies were enhanced chemiluminescence horseradish peroxidase (ECL HRP)-linked rabbit IgG whole antibody (7074) or ECL HRP-linked mouse IgG whole antibody (7076) from Cell Signaling.

### Quantitative real-time PCR

RNA was isolated using RNeasy mini kit (Qiagen). Digested with DNaseI (Sigma-Aldrich) for 15 min at RT to remove DNA contamination, and subsequently, DNaseI was inactivated by heating at 70 °C for 10 min. cDNA was synthesized using Qscript supermix (Quanta Biosciences). Real-time PCR was performed using SSO fast SYBR green supermix (Bio-Rad) in a 7500 real-time PCR system (Applied Biosystems) using 7500 software v2.0.1. Melting curve analysis was performed to ensure the amplification of a single product. Primers used were the following: TCF-1-F-5′-AGGCCAAGAAGCCAACCATCAAGA and TCF-1-R-5′-ACTCTGCAATGACCTTGGCTCTCA; TCF-3-F-5′-TGCAGTGAGCGTGAAATCACCAGT and TCF-3-R-5′-AATGGCTGCACTTTCCTTCAGGGT; TCF-4-F-5′-TCGGCAGAGAGGGATTTAGCTGATGT and TCF-4-R-5′-CTTTCCCGGGATTTGTCTCGGAAACT; LEF1-F-5′-AAGCATCCAGATGGAGGCCTCTACAA and LEF1-R-5′-TGATGTTCTCGGGATGGGTGGAGAAA; β-catenin-F-5′-TCTTGCCCTTTGTCCCGCAAATCA and β-catenin-R-5′-TCCACAAATTGCTGCGTCCCA; EAAT2-F-5′-CCAAGCTTGGATCACTGCCCTGG and EAAT2-R-5′-CCAGCCCCAAAAGAGTCACCCACAA; GS-F-5′-TTGAGAAACTAAGCAAGCGGCACC and GS-R-5′-ATCCAGTTAGACGTCGGGCATTGT; and GAPDH-F-5′-CTTCAACGACCACTTTGT and GAPDH-R-5′-TGGTCCAGGGGTCTTACT. Fold change in messenger RNA (mRNA) expression was calculated by relative quantification using the comparative *C*_*T*_ method with GAPDH as the endogenous control.

### Chromatin immunoprecipitation (ChIP)

PDAs were grown for 24–48 h and ChIPed for the presence of TCF-1 and β-catenin on the endogenous promoter region of EAAT2 or GS using a Millipore kit with antibodies for TCF-1, TCF-3 (anti-mouse, Cell Signaling; 9383), and β-catenin (anti-rabbit, Sigma-Aldrich; C 2206). A suitable mouse IgG1 isotype control (5415) or rabbit IgG control (3900) from Cell Signaling was included in the experiments. Similarly, ChIP was performed on PDA cells transfected with EAAT2-prom for 24 h using anti-TCF-1 antibody. Per immunoprecipitation, approximately 2 × 10^6^ cells and 5 μg of antibody were used. Samples were analyzed by real-time PCR as explained earlier. Primers used to amplify EAAT2 promoter regions were divided into three regions: proximal (F-AAGACACACACTTACCCTTGACGG and R-CGTCTTAGGGCATTTGACTTTGGG), middle (F-TGTGGCCCTCCAAGTGAGTTCTTT and R-TGACGAGACCTGTGCAGCTTTGAT), and distal (F-GGGTGATGTCAGCTCTCGACGAA and R-AGGGAGGGATTGCAAGGTTTAGC). Primers used to amplify the putative GS promoter region were GS-F-TCTCTTGATGGTGCTGCTGTCACT and GS-R-AGAACGGAATGGTAAGGCGTGAGT. Data was normalized to IgG and represented as fold change with respect to IgG.

### Glutamate uptake assay

Cells either transfected with plasmids for 24 h or siRNAs for 72 h were spiked with 2 mM L-glutamic acid (glutamate) and incubated for 30 min, and the final concentration of glutamate in the supernatant was measured by glutamate assay kit (Biovision) as per instructions. A standard curve was generated by diluting the provided glutamate standard in complete DMEM media.

### Vivo-morpholino injection in mice

Animal studies were conducted with approval from the Rush University Medical Center Institutional Animal Care and Use Committee (IACUC). Male 10C57bl/6J mice aged 4–6 weeks were purchased from Jackson Laboratories. Approximately 1 week after arrival, animals were anesthetized with ketamine/xylazine (90 mg/kg, 5 mg/kg, I.P.) and placed in a stereotaxic apparatus. Bilateral injections of vivo-morpholinos (Gene Tools, LLC) were targeted to the mPFC with coordinates relative to Bregma +2.0 A/P, +0.3 M/L, −1.0 D/V. The antisense (AS) sequences for mouse β-catenin was designed from the larger of the two mRNA variants, one near the 5′ region (AS) 5′-GCCGCACAAGGAGCGATTTATAAGC-3′ and one spanning the translational start site (AS2) 5′-CTTGAGTAGCCATTGTCCACGCAGC-3′. A control morpholino sequence 5′-CCTCTTACCTCAGTTACAATTTATA-3′ or PBS was injected as a control. All morpholinos were at a concentration of 500 nM per 1.0 μl. Mice received 1 μl/hemisphere microinjections on day 1 and day 3. Mice were sacrificed by CO_2_ inhalation followed by rapid decapitation 4 days following the second surgery, and brain punches were isolated from the region targeted with the injections. Punches were homogenized in RIPA buffer, and western blots were run as described above.

## Results

### β-Catenin regulates EAAT2 mRNA and protein expression in PDAs

**Fig. 1:**
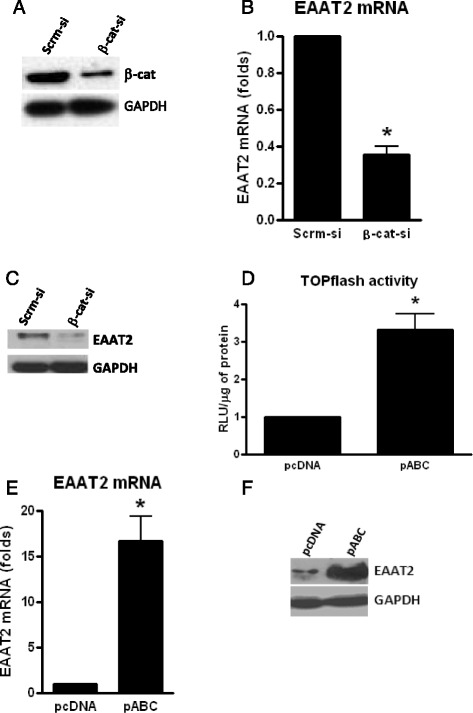
β-Catenin positively regulates EAAT2 mRNA and protein expression in PDAs. PDAs were transfected with siRNA for β-catenin or scrambled siRNA (Scrm-si), and 72 h post transfection, total β-catenin protein was monitored by WB (**a**), EAAT2 mRNA level was monitored by real-time qPCR (**b**), and EAAT2 protein expression monitored by WB (**c**). PDAs were transfected with a plasmid containing a constitutively active form of β-catenin (pABC) or a control plasmid and 24 h later transfected with a TCF/LEF reporter plasmid (TOPflash) and luciferase activity was measured 24 h later (**d**). PDAs were transfected with pABC or control vector (pcDNA) (**e**, **f**), and 24 h post transfection, EAAT2 mRNA (**e**) and protein (**f**) levels were monitored by qPCR and WB, respectively. *Asterisks* indicate a *p* < 0.05 relative to the respective control using Student’s *t* test. *Error bars* indicate SD

We performed our studies in human progenitor-derived astrocyte (PDAs). PDAs exhibit prototypical characteristics of astrocytes including expression of GFAP, EAAT2, and glutamate synthetase and are capable of glutamate uptake in a standard glutamate uptake assay [14]. To assess the impact of β-catenin on EAAT2 gene expression, PDAs were treated with smart pool β-catenin siRNA or scrambled siRNA for 72 h. We typically achieve ~70–80 % β-catenin knockdown (KD), as previously described. KD of β-catenin (Fig. 1a) led to a ~70 % reduction in EAAT2 mRNA (Fig. 1b) and protein expression (Fig. 1c). We also performed gain of function studies using a constitutively active plasmid of β-catenin (pABC). PDAs transfected with pABC for 24 h before TOPflash transfection (containing several TCF/LEF binding sites linked to firefly luciferase as indicator of integrity of β-catenin signaling) induced TOPflash activity after 24 h by approximately threefolds in comparison to backbone vector (Fig. 1d), confirming its ability to over-express β-catenin. Transfection of PDAs with pABC for 24 h also induced EAAT2 mRNA (>15-folds; Fig. 1e) and protein expression (Fig. 1f). Together, loss and gain of function studies indicate that β-catenin positively regulates EAAT2 expression in PDAs.

### β-Catenin partners with TCF-1 to positively regulate EAAT2 expression

**Fig. 2:**
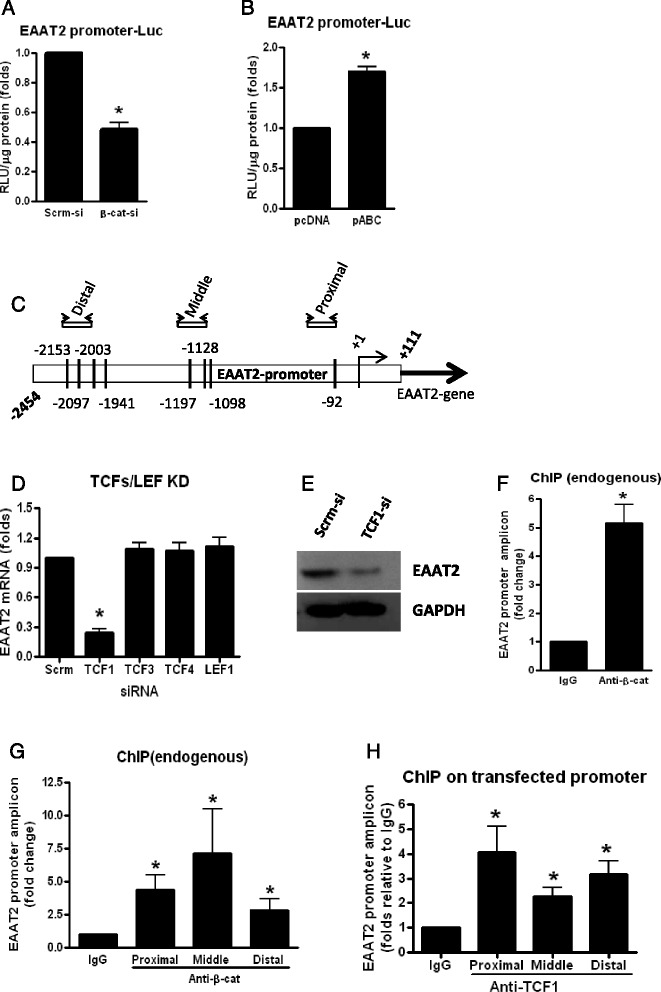
β-Catenin partners with TCF-1 to positively regulate EAAT2 at the level of transcription in PDAs. PDAs were transfected with β-catenin siRNA or Scrm-Si, and 48 h post transfection, cells were transfected with an EAAT2 promoter linked to luciferase, and 24 h later, luciferase activity was measured (**a**). **b** PDAs were transfected with pABC or pcDNA, and 24 h post transfection, cells were transfected with EAAT2 promoter linked to luciferase, and 24 h later, luciferase activity was measured. **c** Identification of putative TCF/LEF binding sites on the EAAT2 promoter. Shown in **c** is a ~2.5-kb promoter of EAAT2 illustrating TCF/LEF binding sites present in clusters and the regions selected for designing primers for real-time PCR. PDAs were transfected with siRNAs to knockdown TCF-1, TCF-3, TCF-4, LEF1, or scrambled siRNA (**d**, **e**), and 72 h post transfection, real-time qPCR was performed to measure the levels of EAAT2 mRNA (**d**) and protein by WB (**e**). PDAs (~2 × 10^6^ cells) were cross-linked with 1.0 % formaldehyde and sonicated to generate chromatin preparations (**f**–**h**). ChIP was performed using antibodies against β-catenin (**f**, **g**) or TCF-1 (**h**) (5 μg antibody/IP). PDAs were transfected with EAAT2-prom, and 24 h later, ChIP was run (**h**). Data shown are normalized to a nontargeting IgG control and reported as a fold change relative to IgG. *Asterisks* indicate a *p* < 0.05 relative to the respective control using Student’s *t* test. *Error bars* indicate SD

To determine if the ability of β-catenin to induce EAAT2 is mediated at the promoter level, an approximate 2.5-kb human promoter for EAAT2 linked to luciferase was transfected into PDAs. This EAAT2 promoter is well characterized [22]. PDAs were transfected with siRNA for β-catenin or pABC for 48 h then EAAT2 promoter. Twenty-four hours after promoter transfection, KD of β-catenin inhibited EAAT2 promoter activity by 50 %, while over-expression of β-catenin induced EAAT2 promoter activity by 0.7-fold, in comparison to their respective controls (Fig. 2a, b).

In the canonical Wnt pathway, β-catenin regulates its target genes by partnering with one of its downstream transcription factors such as TCF-1, TCF-3, TCF-4, or LEF1. To determine the partner of β-catenin in regulating EAAT2 gene expression, we performed bioinformatic studies to identify putative TCF/LEF sites on the EAAT2 promoter. Analysis of the 2.5-kb promoter revealed the presence of at least eight TCF/LEF binding consensus sites (CAAAGA) clustered into three regions, which we designated as proximal, middle, and distal regions with respect to the transcription initiation site (Fig. 2c). Previously, we demonstrated that astrocytes, including PDAs, express all four TCFs/LEF1 and all of them can be efficiently KD by siRNA approach [20]. Individual knockdown of TCFs/LEF followed by quantification of EAAT2 transcript by real-time qPCR after 72 h in PDAs revealed that only loss of TCF-1 inhibited expression of EAAT2 mRNA by 75 % (Fig. 2d). Further, western blot analysis of scrambled and TCF-1 knockdown samples indicated similar results of EAAT2 expression at the protein level (Fig. 2e). ChIPing with β-catenin antibody followed by qPCR for EAAT2 endogenous promoter indicated a strong binding of β-catenin on EAAT2 promoter (Fig. 2f). Further analysis revealed that β-catenin significantly binds to endogenous EAAT2 on all three regions of the promoter compared to IgG (Fig. 2g). However, similar ChIPing strategy did not work for TCF-1 (data not shown). This could be because TCF-1 antibody may be weak and hence may not be pulling down sufficient quantities of genomic DNA for amplification. In order to circumvent this challenge, we transfected EAAT2-prom into PDAs and then performed ChIP assays with anti-TCF-1 and control IgG antibodies. We found significant binding of TCF-1 on all three regions of the promoter compared to IgG, although binding of TCF-1 was higher at the proximal region to levels comparative of β-catenin binding over IgG control compared to the middle or distal regions of EAAT2 promoter (Fig. 2h). These data demonstrate that β-catenin interacts with the TCF-1 bound on the EAAT2 promoter and thereby directly regulates the transcription of EAAT2. Although other members of the TCF/LEF family (TCF-3, TCF-4, LEF1) may be binding to the EAAT2 promoter, given that knocking them down did not alter EAAT2 mRNA levels (Fig. 2d) suggests that even if they bind to the promoter, they are not key players in EAAT2 gene regulation whereas TCF-1 is.

### β-Catenin positively regulates GS expression by partnering with TCF-3

**Fig. 3:**
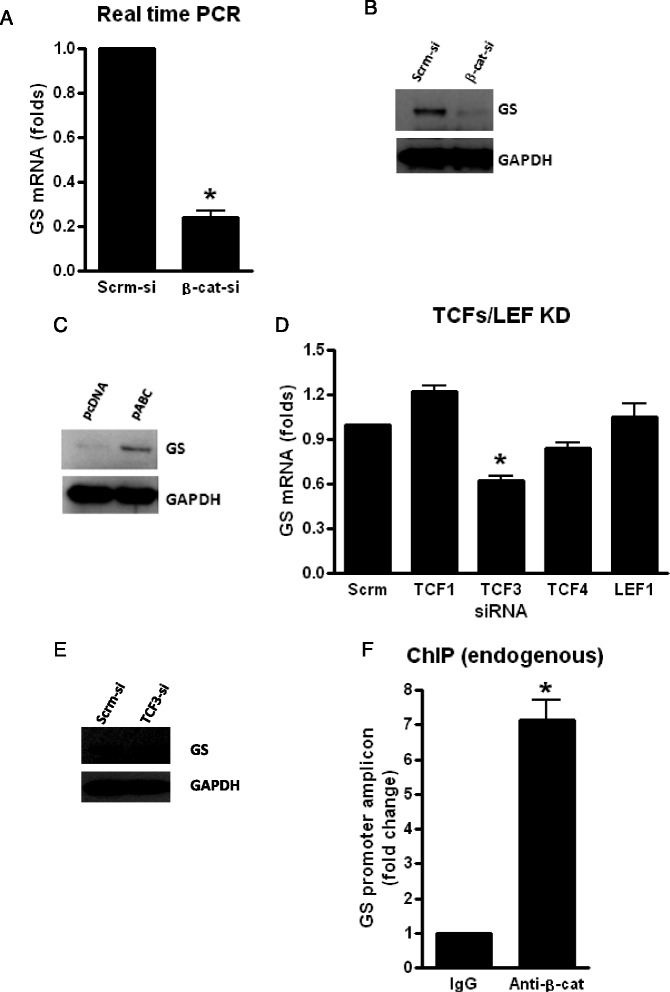
β-Catenin partners with TCF-3 to regulate GS expression at the transcription level in PDAs. PDAs were transfected with siRNA for β-catenin or Scrm-si for 72 h (**a**, **b**) or pABC and pcDNA for 24 h (**c**) and GS mRNA (**a**) and protein (**b**, **c**) measured by real-time qPCR and WB, respectively. PDAs were transfected with siRNA designated for TCF/LEF family members or Scrm-si (**d**), and at 72 h post transfection, GS mRNA (**d**) and protein (**e**) levels were measured by qPCR and WB, respectively. **f** PDAs (~2 × 10^6^ cells) were cross-linked with 1.0 % formaldehyde and sonicated to generate chromatin preparations. ChIP was performed using antibodies against β-catenin (5 μg antibody/IP). Data shown are normalized to a nontargeting IgG control and reported as a fold change relative to IgG. *Asterisks* indicate a *p* < 0.05 relative to the respective control using Student’s *t* test. *Error bars* indicate SD

In order to assess the role of the β-catenin pathway in regulating the expression of GS, we KD β-catenin in PDAs for 72 h and evaluated the expression of GS at the mRNA and protein levels. We show that β-catenin KD significantly inhibited the expression of GS mRNA (~80 %) and protein (Fig. 3a, b) whereas over-expression using pABC for 24 h induced GS protein levels (Fig. 3c). Interestingly among TCFs/LEFs, knockdown of TCF-3 and not TCF-1 for 72 h had a significant effect on the inhibition of GS mRNA (~40 %) and protein expression (Fig. 3d, e). ChIP with anti-β-catenin antibody in PDAs followed by qPCR quantification of endogenous promoter region of GS resulted in approximately sixfold higher pull down of GS promoter region compared to IgG, suggesting a strong binding affinity for β-catenin on GS promoter (Fig. 3f). Collectively, these data demonstrates that β-catenin regulates GS in part through binding to TCF-3 in human astrocytes.

### β-Catenin pathway regulates EAAT2/GS expression and glutamate uptake

**Fig. 4:**
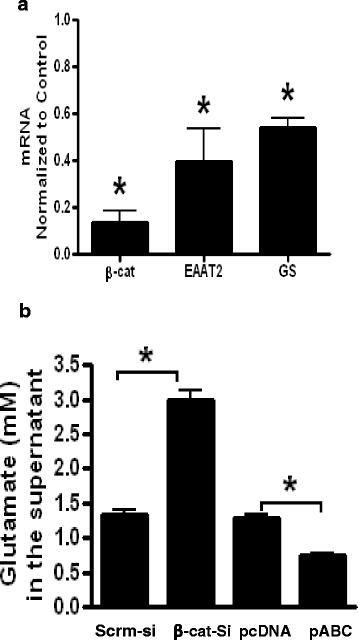
NHAs were transfected with siRNA for β-catenin or Scrm-si for 72 h and mRNA for β-catenin and EAAT2, and GS was measured by qPCR and normalized to control (**a**). **b** PDAs were KD or over-expressed for β-catenin using siRNA and pABC and equivalent controls as indicated previously. At 72 h post β-catenin KD or 24 h post β-catenin over-expression, 2 mM of L-glutamic acid was added to the cell media and 30 min later glutamate in the supernatant was measured using a glutamate assay kit. *Asterisks* indicate a *p* < 0.05 relative to the respective control using Student’s *t* test. *Error bars* indicate SD

β-Catenin regulation of EAAT2 and GS is not limited to PDAs. Using an additional primary human astrocyte, we show that β-catenin KD by siRNA in NHAs resulted in a significant reduction in EAAT2 mRNA by 60 % and in GS mRNA by 50 % (Fig. 4a). Given the significant positive regulation of β-catenin on EAAT2 and GS expression at the transcriptional level, we determined whether modulation of cellular β-catenin levels changes uptake capacity of glutamate in astrocytes. We KD or over-expressed β-catenin in PDAs, spiked the media with glutamate for 30 min, and assessed for glutamate uptake by measuring residual glutamate in the medium. β-Catenin KD led to a twofold increase in glutamate whereas β-catenin over-expression resulted in reduction in glutamate by 50 % in the media (Fig. 4b). These data indicate that β-catenin KD impairs glutamate uptake and conversely its over-expression enhances glutamate uptake.

### β-Catenin regulates expression of GLT-1 and GS in vivo

**Fig. 5:**
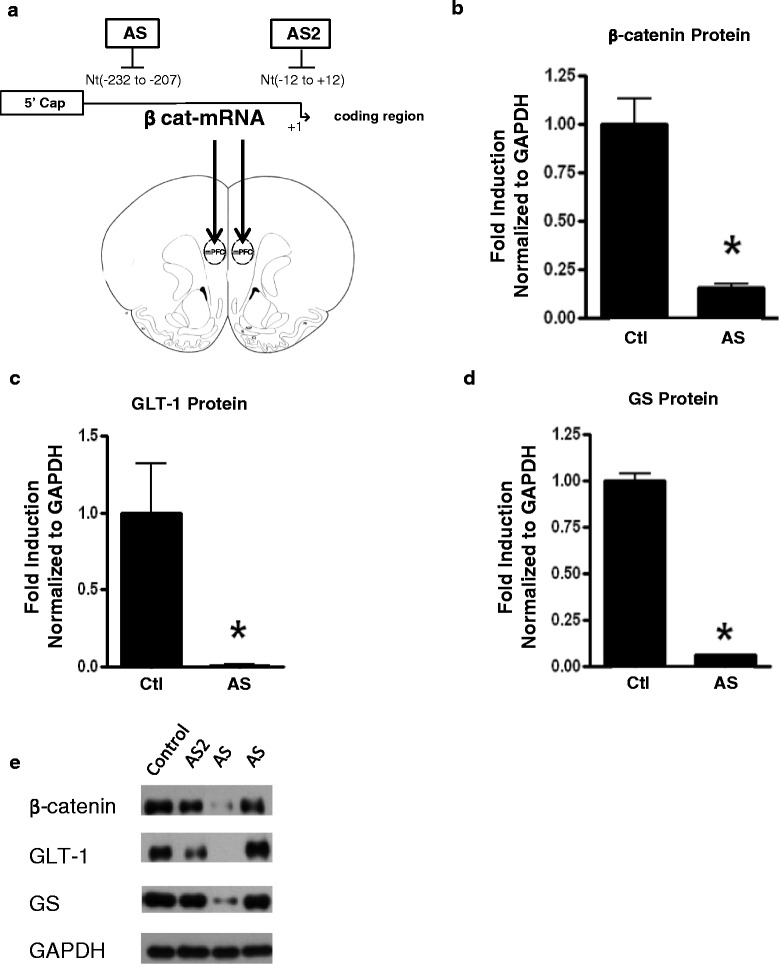
β-Catenin positively regulates GLT-1 and GS in vivo. Male 10C57bl/6J mice were microinjected with control, antisense (AS), or AS2 vivo-morpholinos against β-catenin on days 1 and 3 into the mPFC (**a**). On day 7, mice were sacrificed and brain harvested and β-catenin (**b**), GLT-1 (**c**), and GS (**d**) protein levels were measured by WB in AS treated with a KD of β-catenin. The panel (**e**) is a representative WB of animals treated with control, AS2, AS with KD of β-catenin, and AS without KD of β-catenin. *Asterisks* indicate a *p* < 0.05 relative to the respective control using Student’s *t* test. *Error bars* indicate SD

We next sought to determine whether β-catenin regulates GLT-1 (EAAT2) and GS expression in vivo. Vivo-morpholinos (Gene Tools, LLC) were designed to block translation initiation of β-catenin in the cytosol (Fig. 5a). Mice (10C57bl/6J at 4–6 weeks of age) were given microinjections of AS, AS2, or control into the mPFC on days 1 and 3, and brains were harvested on day 7, and expression of β-catenin, GLT-1, GS, and GAPDH were measured by western blot. AS induced β-catenin KD near the site of injection in most animals except for one animal where no change in β-catenin expression was observed. AS2 had no effect on β-catenin expression in all animals tested. There were no differences of protein expression between animals treated with control morpholino or saline; thus, they were grouped together. Among animals displaying a significant KD of β-catenin compared to control, GLT-1 and GS protein expression was reduced by 95 and 90 %, respectively, in comparison to control animals (Fig. 5e). Among the animals which received AS and did not demonstrate reduction in β-catenin at the sight of injection or animals that received AS2 which were not effective in knocking down β-catenin, no reduction in GLT-1 or GS was observed (Fig. 5e). Together, these data demonstrate that only animals KD for β-catenin have significantly reduced expression of GLT-1 and GS.

## Discussion

The β-catenin signaling pathway is vital to various functions in the CNS ranging from memory consolidation in astrocytes, neurogenesis, and neurotransmitter release to induction of long-term potentiation and depolarization resulting in increased synaptic strengths [21, 23]. Disruption of β-catenin signaling has profound biologic consequences on astrocyte/neuron communication. We previously determined that disruption of β-catenin in astrocytes led to the modification of 128 genes [20]. We showed here, in vitro and in vivo, that disruption of β-catenin leads to dramatic inhibition of the glutamate transport network (EAAT2/GS) within astrocytes.

Through gain and loss of function studies as well as ChIP assays, we demonstrate the molecular mechanism by which β-catenin regulates EAAT2 and GS expression in astrocytes. We show that β-catenin partners with TCF-1 to induce EAAT2 expression and partners with TCF-3 to induce GS gene expression. Although we show that TCF-1 binds to EAAT2 promoter and regulates its expression, other members of TCF/LEF could also be binding to this promoter. In regulating GS gene expression, β-catenin may partner with additional transcriptional factors in addition to TCF-3. This is partly because, although we observed a statistically significant decrease in GS mRNA expression in TCF-3 KD astrocytes, this KD was only 35–40 % less than the control suggesting that other transcription factors may also be involved.

Our in vivo studies also confirmed that β-catenin positively regulates EAAT2/GLT-1 and GS expression. We used vivo-morpholinos to KD β-catenin in the prefrontal cortex of mice. Vivo-morpholinos are well established to effectively KD proteins of interest in many target organs in small rodents [24–26]. However, there are few studies of vivo-morpholinos injected into the brain. We tested two morpholinos for their ability to efficiently KD β-catenin. Of the two morpholinos tested, only AS provided a high level of β-catenin KD resulting in decreased GLT-1 and GS expression. Interestingly, animals injected with the AS2 or animals that received AS but did not have KD of β-catenin had normal expression of GLT-1 and GS. Potential reasons why KD of β-catenin was not observed in the animal with AS could be that the tissue punch was not on the injection site or failure of the morpholino to KD β-catenin in that animal. Regardless, animals KD for β-catenin had subsequent KD of GLT-1 and GS to confirm our in vitro finding that β-catenin positively regulates EAAT2/GLT-1 and GS expression. Since vivo-morpholinos are not cell specific, treatment likely KD β-catenin expression in many CNS cell types. As such, an indirect result of β-catenin knockdown in neurons or microglia could potentially induce the changes in GLT-1 and GS expression through an unexplored mechanism. While this is possible given the vast number of gene targets for β-catenin that remain to be studied in all cell types, our in vivo data substantiates our in vitro findings which strongly supports astrocytic β-catenin transcriptional regulation of EAAT2 and GS protein expression. Further, GLT-1 and GS are predominately located in astrocytes suggesting astrocytic regulation of these proteins which are necessary for glutamate cycling and transmission.

The finding that β-catenin positively regulates EAAT2 and GS has a broader applicability in understanding mechanisms of neurodegenerative diseases which can be related to chronic neuroinflammation. The role of astrocytic β-catenin in neuroinflammation has not been well characterized. Total β-catenin has been shown to be dysregulated in a number of neurodegenerative diseases including Alzheimer’s disease, Parkinson’s disease, amyotrophic lateral sclerosis, and neuroAIDS, in neuropsychiatric disorders such as schizophrenia as well as a decline in Wnt/β-catenin signaling due to aging processes [21, 27–30]. Emerging studies have found a decrease in Wnt/β-catenin signaling in aged rats with an increase in neuroinflammation [27]. Also, agents used to increase Wnt/β-catenin signaling have been shown to be neuroprotective in a Parkinsonian rat model by reducing glial activation and oxidative stress [31]. Interestingly, Wnts, which can be released by astrocytes, have also been linked to inflammatory pathways and neurorepair in Parkinson’s models [32]. Further, dysfunction in the glutamate/glutamine cycle, mostly through reduction or impairment of EAAT2 function, is linked to neuroAIDS and other neurodegenerative diseases such as ALS, AD, epilepsy, and ischemia/stroke [4, 33–43]. Therefore, signals that diminish β-catenin will also impact EAAT2/GS expression and ultimately glutamate excitotoxicity, a common neuropathologic feature of neuroinflammation and neurodegeneration. Indeed, specific inflammatory signals can lead to the inhibition of β-catenin signaling in astrocytes and a subsequent reduction in EAAT2 and GS expression. We previously showed that interferon gamma (IFN-γ) down-regulates β-catenin signaling, which also leads to alteration in EAAT2 expression [44]. Therefore, regardless of what the trigger(s) for neurodegeneration may be, inflammation is a common feature and this state of inflammation may reduce β-catenin and consequently EAAT2/GS expression, enhancing glutamate-mediated neurotoxicity.

Past strategies to eliminate excess glutamate signaling via antagonizing NMDAR receptor (memantine) have had many side effects [4] with little clinical improvement, if any. Memantine is a low-affinity NMDAR antagonist, and complete blocking of NMDAR (N-methyl-D-aspartate receptor) will likely have detrimental effects in the brain. Over-expression of β-catenin can be used as a strategy to induce EAAT2 and GS expression in astrocytes to overcome excess glutamate and neuroinflammation in neurodegenerative diseases. There are a number of small molecules, mostly from the cancer research field, that can induce β-catenin activity [45]. Such molecules can be explored for inducing EAAT2 and GS in the CNS. However, due to the many functions of β-catenin in cell homeostasis, targeting β-catenin for any type of therapeutic intervention will have to be carefully examined to avoid off-target effects. Nonetheless, enhancing glutamate clearance and metabolism remains a viable treatment strategy against excitotoxicity especially due to limited efficacy of glutamate receptor antagonists. A better understanding of glutamate cycling regulation including β-catenin signaling may lead to therapies to ameliorate or reduce neuropathology of numerous diseases linked to glutamate-induced excitotoxicity.

## Conclusions

In summary, we demonstrate that β-catenin signaling in primary human astrocytes regulates key proteins of glutamate/glutamine cycling. β-Catenin partners with transcription factor TCF-1 to positively regulate EAAT2 protein expression. Further, β-catenin KD decreases GS in part through partnering with TCF-3 although there are likely other transcriptional binding partners as TCF-3 KD resulted in a partial decrease in GS expression. Using vivo-morpholinos to KD β-catenin expression in the prefrontal cortex of mice, we show that KD of β-catenin results in KD of GLT-1/GS. By showing here that β-catenin regulates glutamate/glutamine cycle, our findings can now be exploited as a novel and viable target to induce EAAT2/GS on astrocytes and reduce excess glutamate potentially diminishing neuroinflammation/neurodegeneration.

## Abbreviations

ChIP: Chromatin immunoprecipitation
CNS: Central nervous system
EAAT: Excitatory amino acid transporters
EGF: Epidermal growth factor
GS: Glutamine synthetase
GSK3β: Glycogen synthetase kinase 3β
IFN-γ: Interferon gamma
LEF: Lymphoid enhancer factor
LRP: Lipoprotein receptor-related protein
mPFC: Medial prefrontal cortex
NF-kB: Nuclear factor kappa B
PDAs: Progenitor-derived astrocyte
TCF: T cell factor
